# Rapid Fabrication of Fe and Pd Thin Films as SERS-Active Substrates via Dynamic Hydrogen Bubble Template Method

**DOI:** 10.3390/nano13010135

**Published:** 2022-12-27

**Authors:** Deepti Raj, Federico Scaglione, Paola Rizzi

**Affiliations:** Dipartimento di Chimica and Centro Interdipartimentale NIS (Nanostructured Interfaces and Surfaces), Università di Torino, Via Pietro Giuria 7, 10125 Torino, Italy

**Keywords:** thin films, Fe, Pd, DHBT, SERS

## Abstract

Fe and Pd thin film samples have been fabricated in a rapid fashion utilizing the versatile technique of dynamic hydrogen bubble template (DHBT) method via potentiostatic electrodeposition over a copper substrate. The morphology of the samples is dendritic, with the composition being directly proportional to the deposition time. All the samples have been tested as SERS substrates for the detection of Rhodamine 6G (R6G) dye. The samples perform very well, with the best performance shown by the Pd samples. The lowest detectable R6G concentration was found to be 10^−6^ M (479 μgL^−1^) by one of the Pd samples with the deposition time of 180 s. The highest enhancement of signals noticed in this sample can be attributed to its morphology, which is more nanostructured compared to other samples, which is extremely conducive to the phenomenon of localized surface plasmon resonance (LSPR). Overall, these samples are cheaper, easy to prepare with a rapid fabrication method, and show appreciable SERS performance.

## 1. Introduction

The preparation of materials that have both a high surface area and a precise and controlled morphology is of vital importance in many applications, in particular in sensors and in the field of catalysis in general [1,2,3]. The dynamic hydrogen bubble template (DHBT) method is quite attractive and has been proven to be excellent in that regard; it generates clean and porous structures in an efficient and rapid way in the absence of inorganic and organic templates [2]. In addition, it is highly controllable and “green” [4]. In DHBT, the working electrode is subjected to a cathodic potential that is within the H_2_ evolution range. The electrode surface experiences both H_2_ evolution and metal ion reduction simultaneously. Continuous gas nucleation, coalescence, and desorption at the electrode surface results in a self-supporting metallic thin film composed of nanoporous features. The size and the rate of evolution of hydrogen bubbles act as a dynamic template for depositing metal atoms, thus, generating porous materials with 3D “honeycomb” nanostructured architectures [5]. Such materials have found usage in a variety of applications, for example, Li batteries, capacitors, electrocatalysis, superhydrophobicity, and sensing applications [6,7,8,9,10,11,12,13,14,15,16].

Hydrogen gas bubble generation from the electrode surface involves three steps: nucleation, growth, and detachment. Application of high potentials leads to supersaturation of H_2_ close to the electrode, causing heterogeneous nucleation over the entire surface [17]. These bubbles tend to coalesce on the surface and increase in size until the contact angle is so small that they detach. At the same time as this phenomenon, however, the metal is discharging on the electrode surface. The bubbles are insulating, so through a surface-blocking effect, they reduce the effective surface area of the substrate, and therefore, the deposition occurs only in bubble-free areas, as originally observed directly by Tsai and collaborators [18,19]. When the bubbles detach from the surface of the substrate, those areas occupied by the bubbles remain empty. This allows the creation of a surface with a structure very particular, commonly called honeycomb, or the presence of developed dendrites vertically ensuring a high surface area. In fact, these bubbles act as a dynamic template during the process of electrodeposition, hence the name. The pores in the electrodeposited film will have approximately the same size as the diameter of the bubbles at the time of release, with the density of the pores of the film determined mainly by the behavior of the bubbles. The pore size increases with continued deposition because the bubbles have more time to coalesce and grow. The morphology of the metal film will depend on the mechanism of nucleation and metal growth, which depends on hydrodynamic conditions and on the potential applied during electrodeposition. 

Some key parameters can be identified that affect the final structure of the deposited material: potential (or current density); source and concentration of H^+^ (acid or NH_4_Cl); substrate material, shape, and size; presence of surfactants and additives; chemical concentration/solution of metal salts; deposition time; and operating temperature. [20,21,22]. The material of the substrate should not generate too vigorous of an evolution of hydrogen that it does not allow a correct deposition of the metal and prevents the film from growing with an ordered microstructure. Copper is a great option since it guarantees adequate hydrogen evolution, not excessively intense. Cu has been frequently used as a substrate for the preparation of 3D-porous structures along with other metals [23]. It is possible to carry out this electrodeposition, as reported in the literature, also on others surfaces including evaporative films [12], laminae [24], microelectrodes, fine wires, discs [13], or electrodes silk-screened [25] and in all these substrates, the typical honeycomb structured morphology was obtained. The addition of any surfactants or additives can profoundly influence the behavior of the bubbles. There are many reagents that can be added to modify the surface morphology of the electrodeposited metal. Halides are common examples, including NaCl [26], HCl [27], NaBr [28], and NH_4_Cl [29]. Deposition time is also one of the most important variables to take into consideration when synthesizing via DHBT. The material, shape, size, and orientation of the working electrode can also influence the behavior of the bubble. The composition of the electrodeposited film can be readily adapted by altering the operational parameters and concentration of the bath of deposition. Bimetallic surfaces can also be obtained through the deposition of a metal through DHBT on top of a substrate consisting of another metal, which is then prepared by this technique [30]. The DHBT method, therefore, appears extremely interesting because it can quickly and efficiently deposit on both mono- and bimetallic surfaces.

Some of the syntheses and preparations of metal surfaces through DHBT are worth mentioning because they allowed the discovery and evolution of this technique. Co is among the most studied metals with a “honeycomb” structure; much of this work was carried out by Popovic, Nikolìc, and colleagues, which showed clear evidence of the bubble modeling effect of H_2_ [31,32]. Furthermore, Cu has been used extensively as a model for understanding nucleation and metal growth mechanisms via DHBT, leading to a good understanding of its behavior during the deposition [33]. Cherevko’s work demonstrated that honeycomb Ag structures can be easily formed using NH_4_Cl as a source of protons for the formation of H_2_ bubbles instead of an acid [34,35]. This work showed that noble metals could be synthesized with similar macroporous architectures and nanoscale characteristics, such as Cu and Ni, thus, generating desirable materials for a wide variety of applications. Depositions of other metals via DHBT were also investigated, such as Au [36], Pd [37], Ru [38], Bi [39], and Pt [12]. M. Asnavandi and C. Zhao used DHBT electrodeposition to prepare thin Ag, Pd, and Ni films [40]. A platinum film with the typical honeycomb structure was also prepared [41]. Cherevko and collaborators also studied the electrodeposition of Pb, while Huang and collaborators studied porous Sn [42,43].

Surface-enhanced Raman scattering is an incredibly useful technique that involves significant enhancement of Raman signals of analyte molecules adsorbed on nanostructured metal surfaces called substrates [44]. Although the mechanism behind the SERS enhancement has been associated with both electromagnetic and chemical effects, the former is much more widely acknowledged as the dominant effect. When the frequency of the incident electromagnetic radiation matches with that of surface conduction electrons (called surface plasmons) of the nanostructured plasmonic substrate, collective electron oscillations occur which cause an increase in the local electric field around sharp tips, edges, nanopores and interparticle gaps of the substrate—this phenomenon is called localized surface plasmon resonance [45]. Resultantly, it leads to enhancement of the Raman signals [46,47,48]. The nanostructured surface also gives rise to “hotspots”, i.e., places of giant electric field enhancement, which amplify the Raman signals to extremely high levels [49,50]. SERS offers diverse potential applications, including environmental monitoring, bio-analyses, forensic analyses, food quality control, material characterizations, etc. [51,52,53,54,55]. Credited to its remarkable sensitivity and enhancement factor as high as 10^11^, SERS technique can enable single-molecule level detection [56,57,58]. Cheap and convenient portable Raman spectrometers have been coupled with commercial SERS substrates, making SERS a promising analytical technique [44,59,60,61]. An array of nanomaterials with tailored morphologies have been utilized as efficient SERS substrates, credited to several decades of research and advancement in nanotechnology [62,63]. Yet the development of highly sensitive SERS substrates remains crucial and vital to widen the utility of SERS further.

Roughened precious metal surfaces, i.e., Ag, Au, and Cu, have been established to show strong SERS enhancement. But a lack of other active substrates has severely reduced the range of applications of SERS. Previously, FePd alloy has been reported to show impressive SERS performance with its high refractive index [56,64]. Palladium has good surface stability and finds ample utilization in electrochemistry as catalyst. Prior attempts have been introduced surface roughening on Pd to make it a SERS-active substrate, but more studies are still needed [65,66,67]. On the other hand, Fe is generally considered to be SERS-inactive, but numerous studies have displayed its appreciable activity towards SERS due to the lightning-rod effect, which involves huge electromagnetic enhancement at sharp features or high curvature sites, by virtue of their morphology and density [64,68,69,70,71,72,73,74,75,76]. In addition, Fe is a good choice dictated by economic and sustainability reasons, it is not a critical raw material, being abundantly present on earth and does not represent a risk either for the environment or health. Hence, testing out the individual metals for SERS is the aim of this paper. Monometallic thin films of Fe and Pd with high surface areas have been electrodeposited onto copper substrates via the Dynamic Hydrogen Bubble Template method. The as-deposited films have been characterized through the use of SEM, EDS and X-ray techniques. The as-deposited films have been tested as SERS-active substrates and showed impressive performance.

## 2. Materials and Methods

A copper tape was opted as the substrate of 0.5 mm thickness and 0.6 cm width, cut in different lengths depending on the characterization that would subsequently be performed on each of the samples. These substrates, as observed under the electron microscope, exhibit a surface that is not perfectly smooth. The observed roughness can certainly provide preferential sites for the production of bubbles and therefore affect the final structure of the films. From a practical point of view, this did not present a problem on the microstructures obtained, as will be discussed later with the images collected for the different samples. On one of the two faces (the one where there was an adhesive conductive tape), an insulating tape was applied so as to deposit the films in the same way every time and make the measurement the most reproducible. Once the substrate was prepared, it was washed in deionized water, then in ethanol, and again in deionized water and left to air dry for at least one day before carrying out the deposition. Electrolytic solutions were formulated from information present in the literature. The electrolyte used to deposit iron consisted of 0.1 M ferric sulfate heptahydrate (Fe_2_(SO_4_)_3_·7H_2_O) as the source of iron and 1.5 M ammonium chloride (NH_4_Cl) as a proton source, which is commonly used during electrodepositions via DHBT [77]. To deposit palladium, the electrolyte was composed of 0.1 M tetraamminepalladium chloride [Pd(NH_3_)_4_Cl_2_] and 1.5 M NH_4_Cl. A three-electrode cell setup connected to the Autolab potentiostat (Metrohm, Utrecht, The Netherlands) was used to prepare the samples. Platinum grid acted as the counter electrode, Ag/AgCl electrode as the reference in double-bridge configuration loaded with saturated solution of KCl and the conductive substrate of copper was the working electrode. The substrate was always immersed in the electrolyte solution vertically, reaching the maximum possible depth (without touching the bottom of the beaker). Electrodeposition was achieved at a constant potential of −4 V. The deposition times were 90, 180, and 300 s for each electrolyte. All syntheses were carried out at room temperature. Once the deposition was carried out for the predetermined time, the sample was washed in deionized water and left to dry in sample tubes. The as-deposited samples have been referred to as Fe90s, Fe180s, Fe300s, Pd90s, Pd180s, and Pd300s (the number in each name denotes the time of deposition in seconds). Then, the extra part of copper that served as contact with the clamp (therefore, not immersed in the electrolytic solution) was eliminated along with the initial part of the deposited film (approx. 1 cm) in order to avoid the possibility of nonhomogeneity of the film that can occur in this area that is closer to the solution/atmosphere interface.

The samples were weighed before and after each deposition, and the weight of the deposited film was determined. Since each deposition appeared uniform, the surface density in g/cm^2^ was also calculated for each sample by dividing the measured weight by the area of the substrate. These samples were then subjected to a morphological and structural characterization using SEM (Inspect SEM, FEI) (FEI, Hillsboro, OR, USA) equipped with an EDS probe (Oxford Ultim-Max 100) (Oxford Instruments, Abingdon, UK), a Panalytical X-pert X-ray Diffractometer in Bragg–Brentano geometry (Panalytical, Almelo, The Netherlands), and an Autolab potentiostat (the same as used in the preparation of the samples). The XRD peaks were indexed with the use of X’Pert Highscore software (version 2.2c (2.2.3.)).

A Renishaw inVia Raman Microscope (Renishaw, Wotton-under-Edge, England) with 514 nm laser line was used for SERS measurements, which were carried out in rotating mode [78]. The rotating sample platform allows the sample to be presented to the SERS detector in a continuous manner, minimizing background corrections and sample adjustment time [79]. A small value of standard deviation has been found, which reinforces the fact that the rotating mode allows for more reproducible and consistent measurements. The following conditions of measurements were applied: 20 s acquisition time, 20 × ULWD objective, and 0.095 mW laser power at the sample. Rhodamine 6G (R6G) dye was used as the probe molecule. The sample preparation involved cleaning the substrate in concentrated HNO_3_ for 5 min followed by rinsing with de-ionized water for several times. Then the sample was immersed in solution of Rhodamine 6G dye in de-ionized water of concentrations 10^−3^ M, 10^−4^ M, 10^−6^ M, 10^−8^ M, 10^−10^ M, and 10^−12^ M for 15 min in order to allow the probe molecules to be adsorbed at the sample surface. All solutions were prepared with chemical-grade reagents and de-ionized water. Air-drying and measuring the sample surface followed.

## 3. Results and Discussion

Thin films of pure Pd and Fe were deposited via DHBT electrodeposition. Each of the prepared samples was characterized using SEM, EDS, and XRD techniques. The characterization stage is fundamental to understanding if the properties that will be determined later for the different samples are characteristics of the deposited material or if the quantity of porous film is so low that the measurements were instead performed on the copper substrate.

### 3.1. Copper Substrate

**Figure 1:**
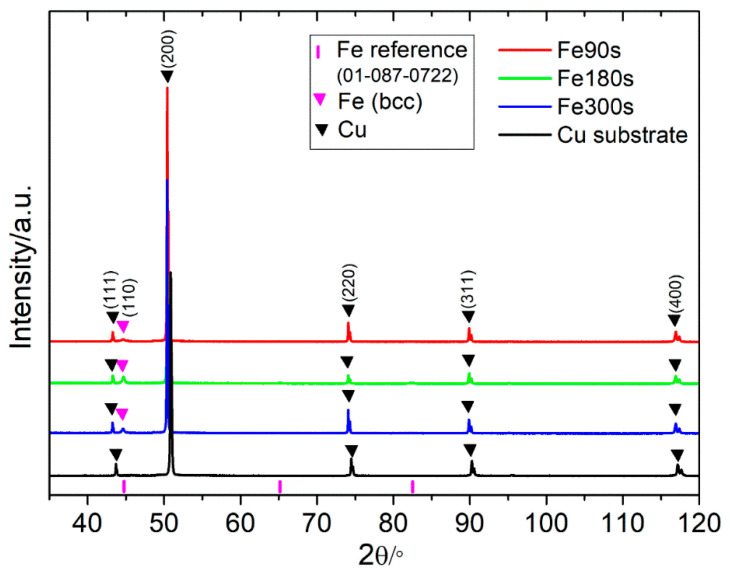
Diffractogram of the different Fe samples obtained by DHBT. The black curve shows the diffractogram of the copper substrate alone.

The surface of the substrate was analyzed through SEM, and from the image in Figure S1 of the supplementary material; it is evident that the copper strip has suffered a lamination process during production to make it thinner. In fact, there are clearly visible lines on the surface of the metallic tape (formed during rolling), all of which are parallel to each other and have a thickness within the micrometer range. The EDS investigation confirmed that the material is composed entirely of Cu (99.98%), confirming the specifications on the commercial conductive tape box. It is also important to record the XRD diffractogram of the copper substrate since in case the thickness of the deposited films via DHBT is not too high, X-rays are often able to penetrate through the film to the substrate and then return to peaks corresponding to the substrate material. Having a diffractogram of a blank copper substrate allows one to obtain an analog to the one in analytical chemistry called “white”, so as to easily distinguish the peaks generated by the Cu substrate from the peaks belonging to the as-deposited material. Figure 1 shows the diffractogram obtained from the copper tape (denoted in black) [80]. By increasing the deposition time, the blank reflections were shifted progressively to the left. This has to be considered an artifact due to the instrumental conditions of the Bragg-Brentano XRD and the thickness of the deposited layers; as the latter increases with the deposition time, the line source no longer remains coplanar with the z-axis of the Cu substrate, and thus, the resulting pattern shows left-shifted peaks. 

### 3.2. Electrodeposition of Iron

**Figure 2:**
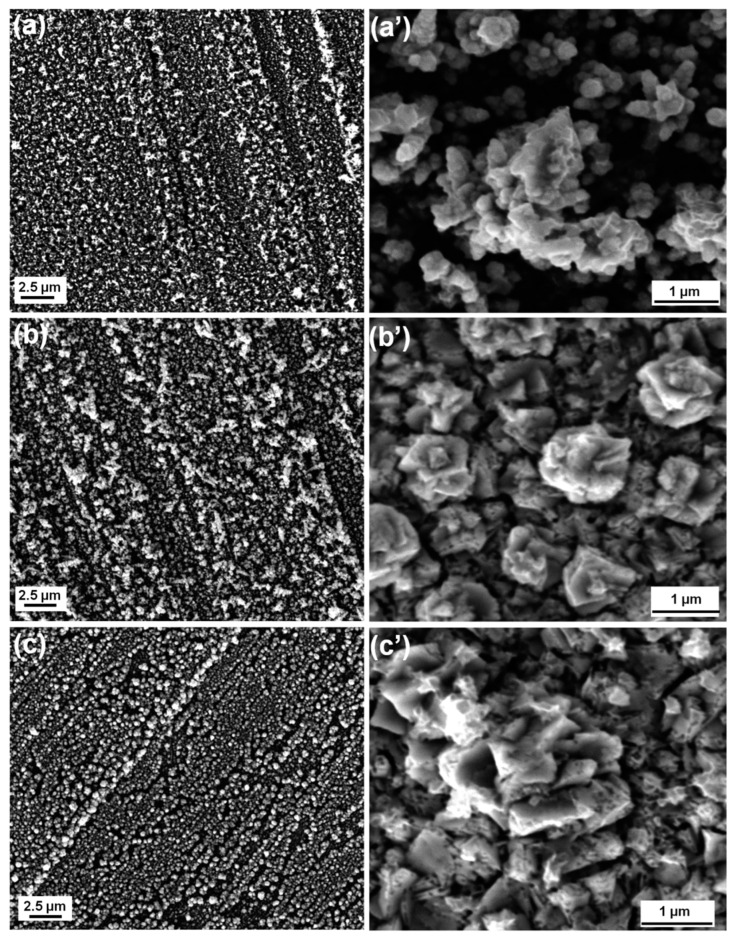
SEM images of the top surface of (**a**) Fe90s, (**b**) Fe180s, and (**c**) Fe300s with their corresponding highly magnified images in (**a’**–**c’**).

Firstly, thin films of Fe have been electrodeposited. As found in the literature, the most common conditions to perform DHBT electrodeposition in a potentiostatic setting involve applying a potential between −3 and −5 V (both potentials are negative enough to get out of the stability range of the solvent, water). Based on this observation, a potential of −4 V was chosen as the deposition potential in this case [81,82]. The potential indeed has a profound influence on the rate of metal deposition at the electrode and on the development of hydrogen, and therefore, on the final structure possessed by the samples. Figure 2 shows the SEM images of the surfaces of the electrodeposited Fe samples. The obtained structure is not of the honeycomb type, and only a small amount of Fe deposition has been achieved. EDS confirmed the presence of Fe, as expected. As the deposition time increases, the microstructure changes; in fact, the images (a) and (b) in Figure 2 show a less compact dendritic-type growth, while in (c), the metal possesses a structure more similar to that of particles deposited on the surface. At higher magnification, the dendritic growth can be observed more closely (Figure 2a’–c’). With the increasing deposition time, the dendrites undergo a gradual development from being in clusters as in the case of Fe90s, to taking up a flower-like shape and morphology seen for Fe180s and eventually acquiring the most defined shape visible in Fe300s. The deposited Fe amount increases with increasing deposition time; this suggests that the longer deposition time results in the substrate acquiring enhanced coverage while a lesser surface area remains exposed to be detected by the beam limited by its depth of penetration.

The XRD diffractograms of the substrate and the as-deposited samples are displayed in Figure 1. The samples show all peaks of Cu with an additional peak of bcc Fe (110) (denoted by the pink triangle). The peak positions of Cu have been shifted to the left as a result of layers of deposited Fe, which increased the overall thickness of the sample. On the other hand, peaks of Fe maintain their position since their position is always the same, i.e., on top of the sample. Other iron peaks are not clearly visible; only the most intense peak is seen, probably because the deposition layer was too thin. Additionally, there are no peaks attributable to iron oxides. Based on these results, it is clear that mainly metallic Fe has been deposited on the copper substrate. 

### 3.3. Electrodeposition of Palladium

**Figure 3:**
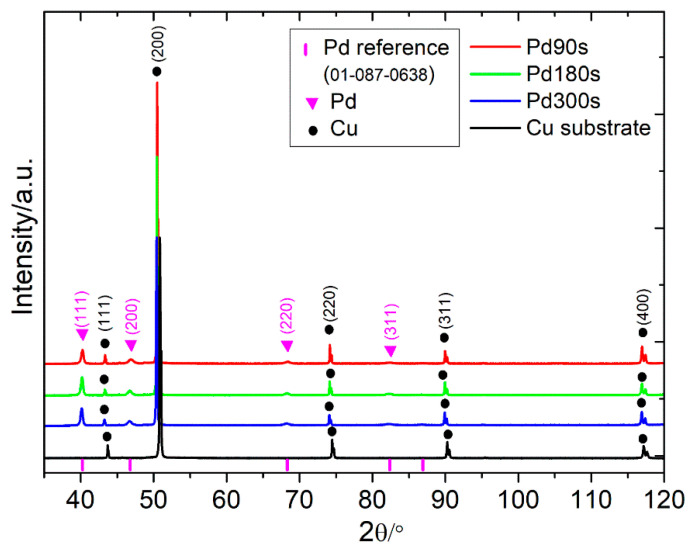
Diffractogram of the different Pd samples obtained by DHBT. The black curve shows the diffractogram of the copper substrate alone.

**Figure 4:**
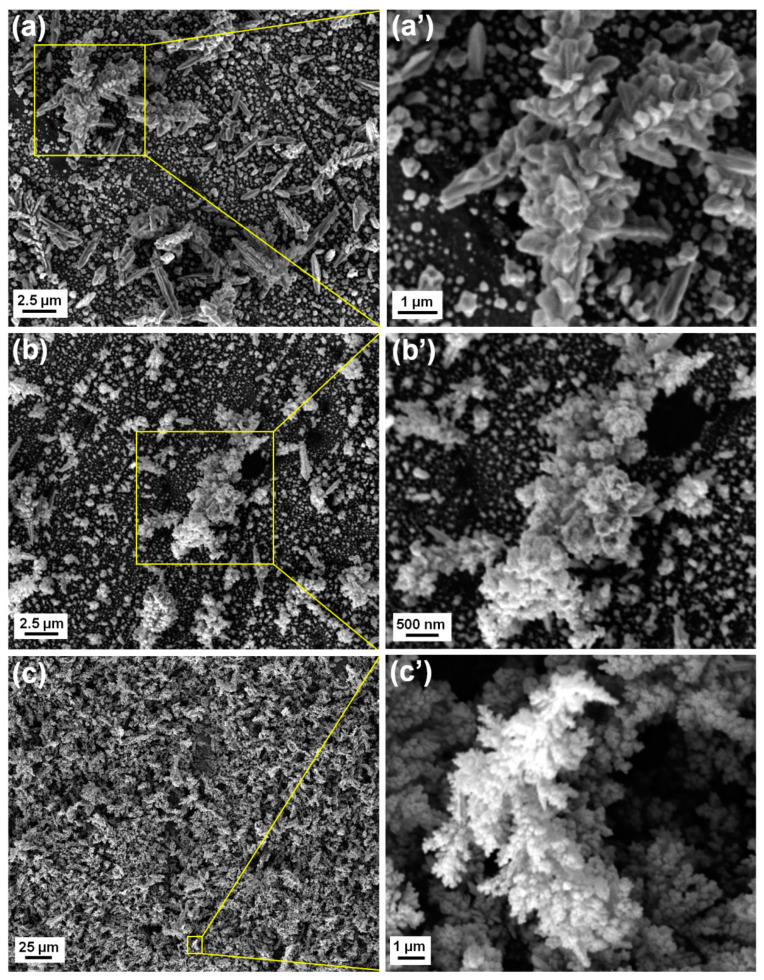
SEM images of (**a**) Pd90s, (**b**) Pd180s, and (**c**) Pd300s while (**a’**–**c’**) provide a closer look at the growing dendrites of the samples, respectively.

**Figure 5:**
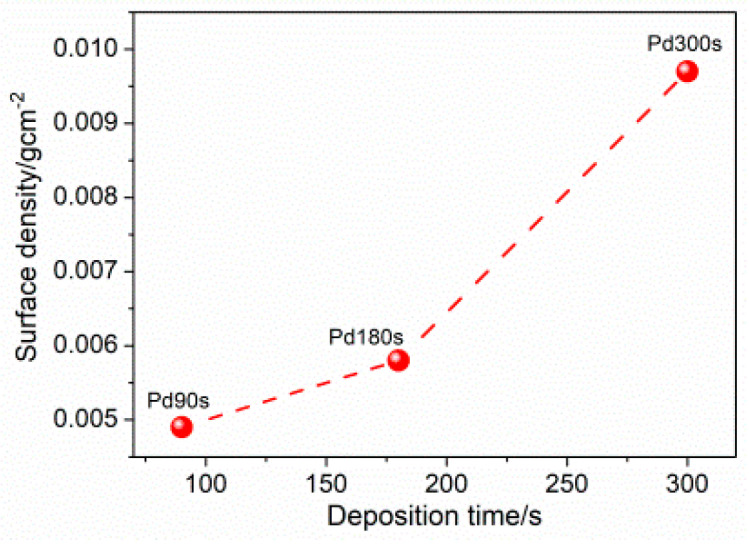
Relationship between the surface density of Pd samples and the deposition times. The dashed line is used as a guide for the eyes.

Figure 3 shows the XRD pattern for the as-deposited samples. The representative peaks for pure Pd are apparent, along with the peaks associated with the Cu substrate. From this diffractogram, it is possible to draw qualitative considerations about the whole. The SEM images obtained for the different samples have been provided in Figure 4. By observing the images from top to bottom in the order of increasing deposition time, it provides the idea that as the deposition time increases, the quantity of metal that is discharged at the electrode is always greater and the coverage becomes progressively dense. This is most evident, particularly in Pd300s, while the differences are less obvious between Pd180s and Pd90s. There is an obvious presence of dendrites in Pd90s (Figure 4a) that are randomly spread over the entire sample surface. With an increase in deposition time, i.e., for Pd180s (Figure 4b), the dendrites continue to grow and start to occupy more surface area. Eventually, in Pd300s (Figure 4c), they can be seen to be ubiquitous throughout the sample surface. Notably, the conditions of deposition do not lead to a well-organized honeycomb-like structure. The SEM images in Figure 4a’–c’ allow seeing the growing dendrite more closely. The morphology of the dendrites appears with a more classic shape with many lateral ramifications. The composition of Pd90s, Pd180s, and Pd300s was checked by EDS, and just Pd was detected predictably. The densities of the materials deposited for Pd90s, Pd180s, and Pd300s were found to be 0.0049, 0.0058, and 0.0097 g/cm^2^, respectively. Figure 5 shows the relationship between the surface density of the Pd samples and the deposition times, and it is clear that the surface density increases with increasing deposition time.

### 3.4. SERS Measurements

**Figure 6:**
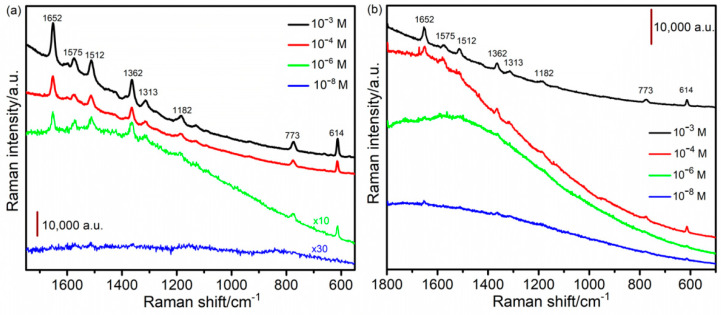
SERS spectra of Rhodamine 6G dye for different concentrations using (**a**) Pd180s (×10 and ×30 stand for the magnification provided in case of 10^−6^ M and 10^−8^ M concentrations to improve the comprehensibility of the spectra) and (**b**) Pd300s as substrates.

**Figure 7:**
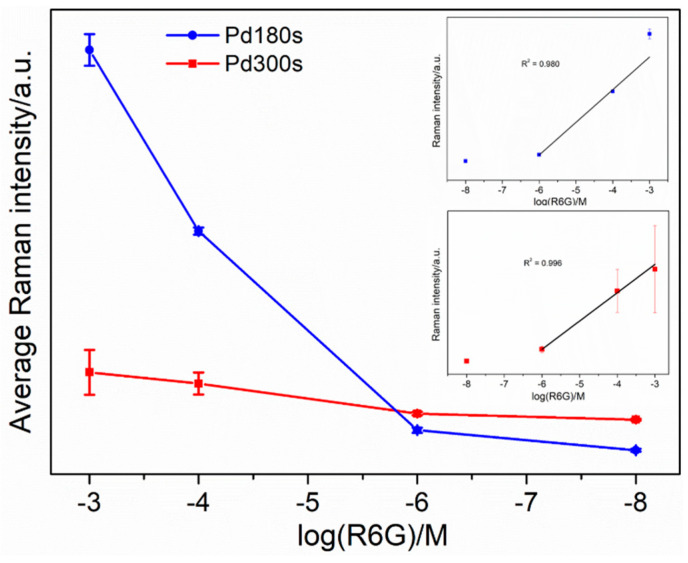
Relationship between the logarithm of R6G concentration and the average SERS intensity at 1652 cm^−1^ and the related error bars. Insets: the SERS intensity at 1652 cm^−1^ as a function of the logarithmic R6G concentrations with the corresponding calibration curve (in black) for Pd180s (top inset) and Pd300s (bottom inset).

The as-prepared thin film samples were employed as a SERS substrate for detection of the probe molecule, Rhodamine 6G dye (R6G). Solutions of R6G in de-ionized water were prepared with different concentrations, i.e., 10^−3^ M, 10^−4^ M, 10^−6^ M, 10^−8^ M, and 10^−10^ M. The samples were immersed in each solution for 15 min, air-dried, and then, subjected to the SERS measurements. Figure 6a,b display the spectra obtained for Pd180s, Pd300s, and Fe180s, respectively. The representative peaks of the probe molecule are shown by all the three mentioned samples at 1652 cm^−1^, 1575 cm^−1^, 1512 cm^−1^, 1362 cm^−1^, 1313 cm^−1^, 1182 cm^−1^, 773 cm^−1^ and 614 cm^−1^ shown. The nanostructured substrate surface with nanogaps between the interconnected dendrites is believed to be the key factor behind the SERS performance attributed to the electromagnetic enhancement effect [83]. In the case of Pd180s (Figure 6a), the signals are very evident at a low concentration of 10^−3^ M until 10^−6^ M, after which the signals are not clear at 10^−8^ M. Hence, 10^−6^ M (479 μgL^−1^) is the lowest possible R6G concentration that could be detected by the sample, Pd180s. This limit of detection is comparable to those previously reported for R6G, e.g., amorphous semiconducting rhodium sulfide microbowl substrates and 2D titanium carbide (MXene) [84,85]. Similarly, Pd300s in Figure 6b continue to show signal enhancement from 10^−3^ M R6G, decreasing in intensity until 10^−8^ M. Despite being a lesser SERS-active metal than Pd, Fe180s reveals appreciable performance with apparent signal enhancement and a low detection limit of 10^−6^ M (as shown in Figure S2b of the supplementary material). Overall, these samples act as rapidly fabricated SERS-active substrates with a low detection limit that are sustainable and comparatively cheaper alternatives to the commercially available substrates. The spectra for the rest of the samples can be found in the supplementary material in Figure S2. Figure 7 depicts a graph of the average Raman intensity vs. the log of concentration of R6G for Pd180s and Pd300s. As expected, the signal gradually decreases in intensity from a higher to a lower concentration. Most notably, the standard deviation is found to be quite marginal, making the rotating mode of the SERS measurement a very reliable path that ensures reproducibility and stability. It also shortens the total time of measurement since it is no longer required to record signals over multiple spots on the sample surface, instead, this mode considers the whole sample surface in each acquisition and generates the average signals. Quantitative analysis of the limit of detection (LOD) was also performed for Pd180s and Pd300s by establishing a calibration curve between the SERS intensities at 1652 cm^−1^ and logarithmic concentrations of R6G (Figure 7 inset). Linear correlations exist between the SERS intensities at 1652 cm^−1^ and the concentrations of R6G ranges from 1 × 10^−6^ to 1 × 10^−3^ M, with an *R*^2^ value of 0.980 and 0.996 for Pd180s and Pd300s, respectively. To calculate the LOD, the following formula was used: LOD = 3.3 × S_b_/b, where S_b_ stands for the standard deviation of the SERS intensity of the blank samples at a Raman shift of 1652 cm^−1^, and b stands for the slope of the calibration curve [86]. The LOD calculated for Pd180s and Pd300s is 1.76 M and 8.2 × 10^−1^ M, respectively. The enhancement factor (EF) was also determined according to the following equation:

EF = (I_SERS_/c_SERS_)/(I_RS_/c_RS_) (1)

where I_SERS_ and I_RS_ stand for Raman intensities of SERS and non-SERS substrates, respectively, while c_SERS_ and c_RS_ are analyte concentrations used for SERS and non-SERS substrates, respectively [76]. The roughly estimated EF value for Pd180s was ~10^3^ which is comparable to those previously reported in the literature, e.g., a single-layer graphene on a 3D Au@Ag [87,88,89,90]. The RSD of the average SERS intensities of different Pd180s substrates from different batches was found to be 8.2%, confirming good substrate-to-substrate reproducibility of the SERS signal [91].

## 4. Conclusions

Fe and Pd films have been potentiostatically electrodeposited at −4 V via the dynamic hydrogen bubble template method. With the increase in the deposition time, a higher quantity of metal is deposited, and the composition of the metal increases as well as the surface density. The microstructure evolves from a dendritic type to nanoparticles in the case of Fe, while for Pd samples, a more classic dendritic shape appears. All the fabricated samples were tried as SERS-active substrates for detection of the probe molecule, rhodamine (R6G). All the samples are able to enhance the Raman signals for R6G showing the representative peaks. Pd180s stands out in terms of its performance, with a low detection limit of 10^−6^ M. Remarkably, these samples are fairly cheaper than reported substrates in literature. Concentrations of the precursor salts of the metal are not required in high quantity to obtain the film, which is a great advantage for economic and environmental sustainability aspects that are increasingly important today. This aspect not only reduces the costs but also makes the overall production less wasteful and environmentally sustainable. 

## Data Availability

Data will be made available on request.

## Supplementary Materials

The following supporting information can be downloaded at: https://www.mdpi.com/article/10.3390/nano13010135/s1, Figure S1: SEM image of the copper substrate; Figure S2: SERS spectra of Rhodamine 6G dye for different concentrations using (a) Fe90s, (b) Fe300s and (c) Pd90s as substrates.
