# Supplementary material for: Rapid Fabrication of Fe and Pd Thin Films as SERS-Active Substrates via Dynamic Hydrogen Bubble Template Method
Source: Nanomaterials (Basel). 2022 Dec 27;13(1):135. doi: 10.3390/nano13010135 (PMC9824498; doi:10.3390/nano13010135)
Supplement: Supplementary file 1 [file nanomaterials-13-00135-s001.zip › nanomaterials-2049673-supplementary.pdf]

nanomaterials

MDPI

*Supplementary Material*

# Rapid Fabrication of Fe and Pd Thin Films as SERS-Active Substrates via Dynamic Hydrogen Bubble Template Method

**Deepti Raj, Federico Scaglione* and Paola Rizzi**

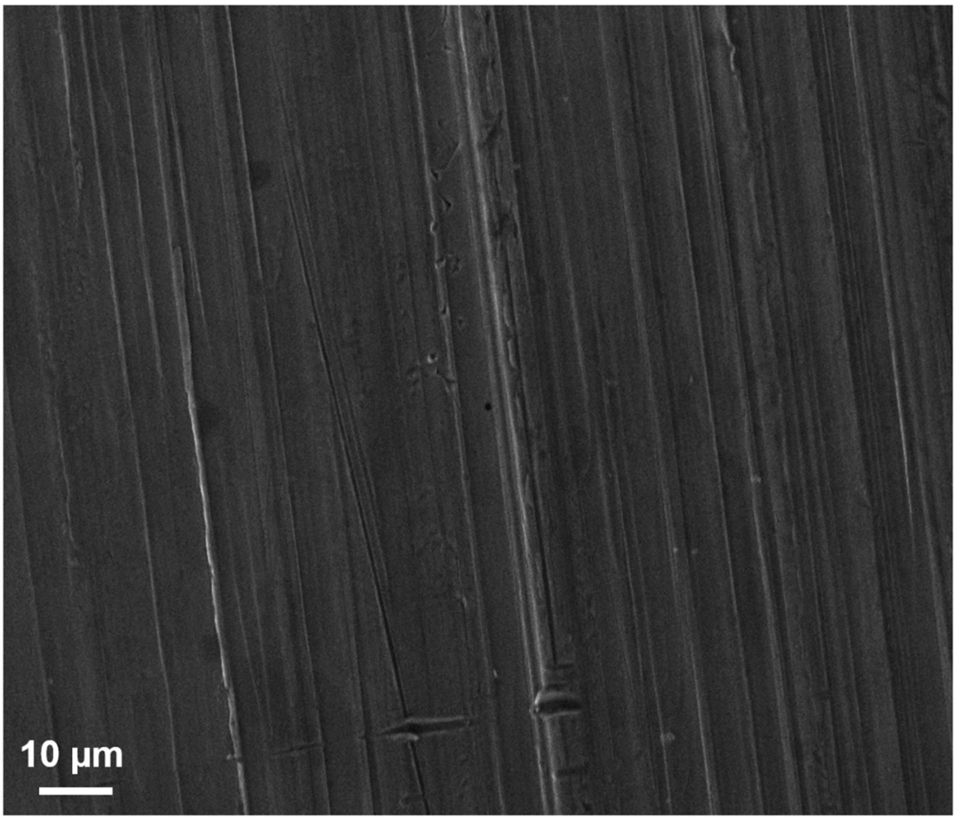


**Figure S1.** SEM image of the copper substrate.

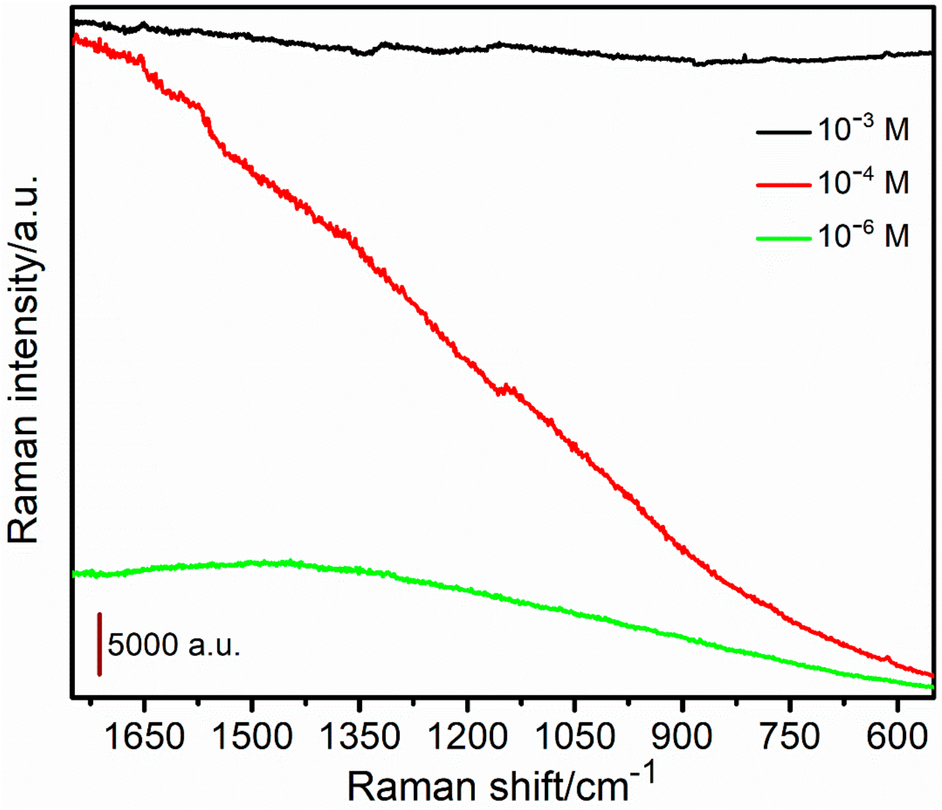

Raman intensity/a.u.
10^-3 M
10^-4 M
10^-6 M
5000 a.u.
1650 1500 1350 1200 1050 900 750 600
Raman shift/cm^-1


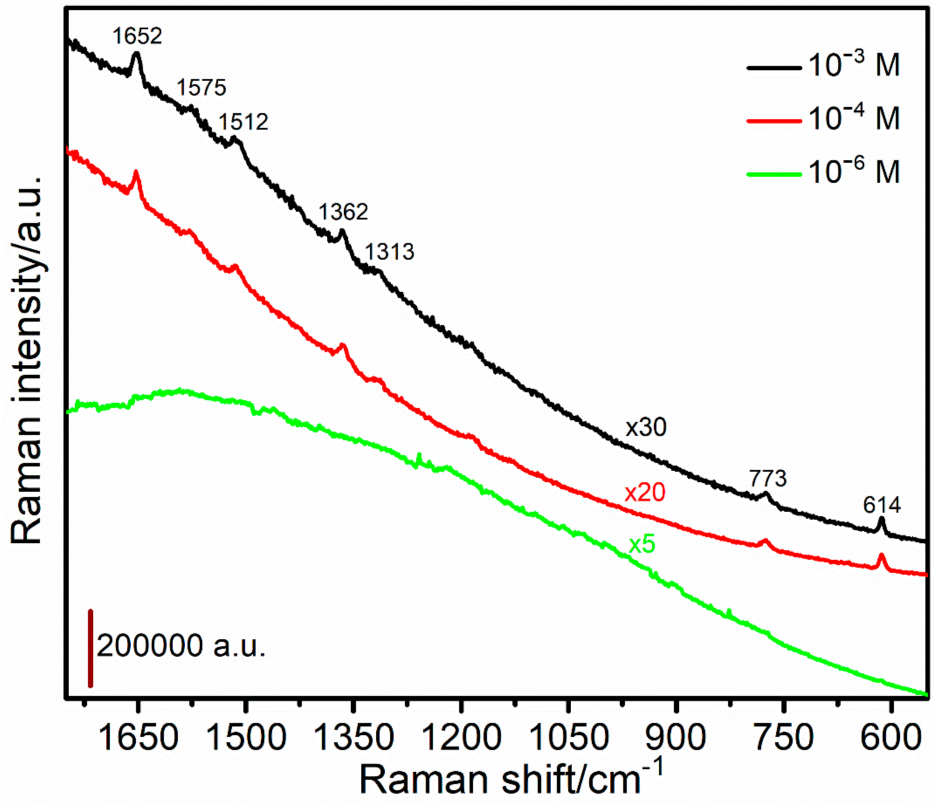

Raman intensity/a.u.
1652
1575
1512
1362
1313
773
614
x30
x20
x5
10^-3 M
10^-4 M
10^-6 M
200000 a.u.
1650 1500 1350 1200 1050 900 750 600
Raman shift/cm^-1

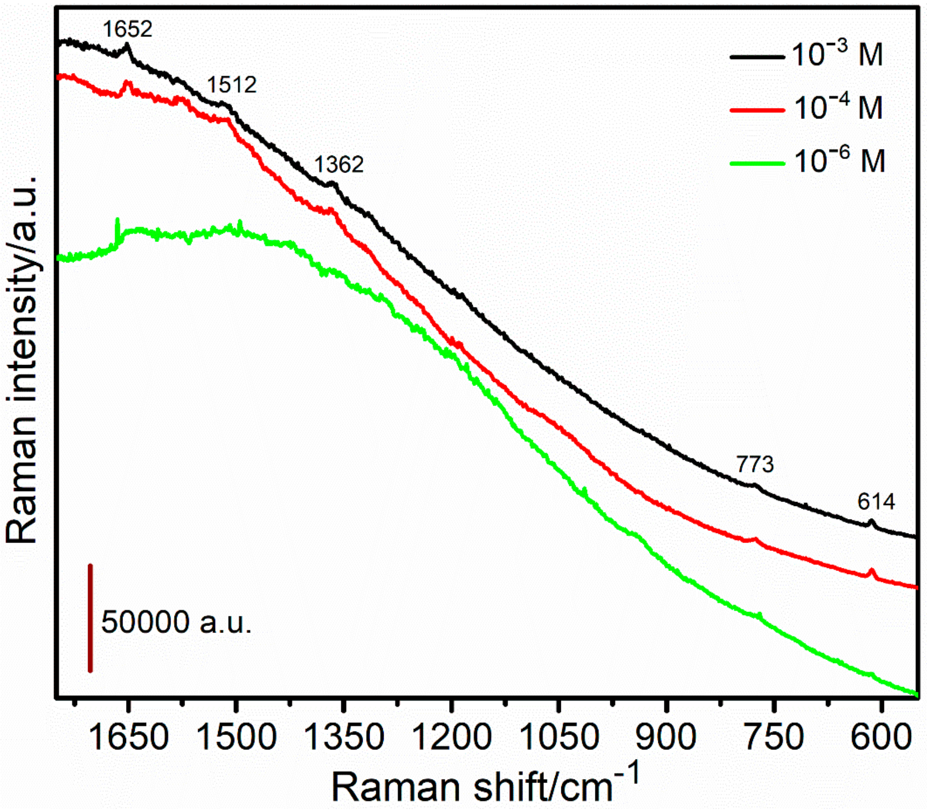


**Figure S2.** SERS spectra of Rhodamine 6G dye for different concentrations using (**a**) Fe90s, (**b**) Fe300s and (**c**) Pd90s as substrates.
